# PH-responsive hydrolysis of salicylate-based polyanhydride copolymer fibers for targeted drug delivery

**DOI:** 10.1177/08839115251410375

**Published:** 2026-02-25

**Authors:** Kenya Whitaker-Brothers, Mohammed Ragib Hasan, Tamima Umme, Kathryn Elizabeth Uhrich

**Affiliations:** 1 Department of Chemical and Biochemical Engineering, Rutgers University, Piscataway, NJ, USA; 2 Department of Chemistry, University of California, Riverside, CA, USA

**Keywords:** polyanhydride, biodegradable, fiber, pH, salicylic acid

## Abstract

Periodontitis is a chronic inflammatory condition that leads to tissue damage, bone loss, and gingival recession. In this disease, pH levels in the mouth are affected by food and saliva as well as tissue inflammation and damage caused by periodontitis. Monitoring pH levels helps assess disease progression and treatment outcomes; therefore, it is important to study the degradation of the biomaterials under relevant pH conditions. Fibers are valuable in treating periodontal disease by enabling site-specific drug delivery to affected soft and hard tissues. In this work, we evaluate polyanhydride fibers that undergo hydrolysis to yield salicylic acid as a function of pH conditions. The physical properties of the polyanhydrides, specifically those based on ester-containing carboxyphenoxydecanoate (CPD), can be altered by copolymerization with ether-containing *para*-carboxyphenoxyhexane (*p*CPH) to affect degradation rates under specific pH conditions. Overall, we observed that the CPD:*p*CPH copolymer is more resistant to hydrolysis than the CPD homopolymer. We examined the effects of degradation media pH on the hydrolysis of 50:50 CPD:*p*CPH fibers (fibers containing 50% CPD and 50% *p*CPH) with pH values ranging from pH 6 to 9. This pH range was chosen as it covers the pH values typically observed in the gingival crevicular fluid (GCF) as well as in the gastrointestinal tract. As a general trend, increasing the pH of the degradation media increased the rate at which the copolymer fibers were hydrolyzed. This work will help enable targeted drug delivery to specific areas in the body, such as acidic tumors or the gastrointestinal tract, for efficient medication release.

## Introduction

For the treatment of periodontal disease, polymer fibers (such as Actisite) are particularly useful for site-specific drug dosing; the fiber is cut and then packed into the defect and/or wrapped around the affected tooth where it locally delivers a drug to treat the infection. Periodontitis is a chronic inflammatory condition that affects the supporting tissues of the teeth and is primarily caused by specific microorganisms, particularly Gram-negative anaerobic bacteria. The condition results in the destruction of the periodontal ligament, bone loss, gingival recession, and the formation of pockets, impacting ~5%–30% of adults.^1^ Along with tissue inflammation and damage caused by periodontitis, pH levels are altered in saliva and blood. Monitoring pH helps assess disease progression and treatment outcomes, thus salivary pH serves as a valuable biomarker for evaluating periodontal health and treatment efficacy.^2^ The pH of the gingival crevicular fluid (GCF) varies as a function of health: pH is roughly 6.5 at healthy sites and varies from pH 6.9 to 8.7, with inflammation.^3^ In addition, the GCF pH may vary from person to person, and even within the same person over the course of the day. Generally, the pH of the oral cavity often lies between 6 and 9, with salivary pH correlating to periodontitis.^2^ Similarly, literature on wound healing suggest that an alkaline pH is conducive to bacterial growth, such that local management of pH may mitigate infection.^4^ Thus, a periodontal treatment that can manage inflammation and infection would be desirable.

With the ability to locally release salicylic acid, salicylate-releasing polymers have been investigated for dental treatments, including implantable membranes for periodontal disease^5–7^ and depot microspheres for deep bone infections.^8^ Several poly(anhydride-esters) based on carboxyphenoxydecanoate (CPD) have been demonstrated to undergo hydrolysis to release salicylic acid,^9^ which is a well-known, anti-inflammatory and antimicrobial analgesic.^10^ Salicylate has several therapeutic functions – keratolytic, anti-inflammatory, antiseptic, analgesic. For this work, the most important aspects of salicylic acid relate to acidity (to create an unfavorable pH environment for bacteria) and its anti-inflammatory effect (to alleviate pain associated with swelling).

The salicylate-based poly(anhydride-esters) are unique because of the relatively high concentration of salicylic acid (~75 wt.%) that can be locally released as the polymer undergoes hydrolytic degradation.^5^ As previously demonstrated for other polyanhydrides,^11–15^ the physical properties of the homopolymer can be modified by copolymerization with monomers such as the ether-containing *para*-carboxyphenoxyhexane (*p*CPH) (3) to increase the hydrolytic stability and mechanical properties of the polymer.^16^ Previous studies demonstrated that copolymers of the salicylate monomer (CPD) and *p*CPH could be prepared at ratios varying from 10 mol.% CPD (written as 10:90 CPD:*p*CPH) to 90 mol.% CPD and melt-extruded to yield solid, continuous fibers.^17,18^ Further, the impact of copolymer composition on the erosion mechanism and mechanical properties of copolymer fibers were investigated.

**Scheme 1.**
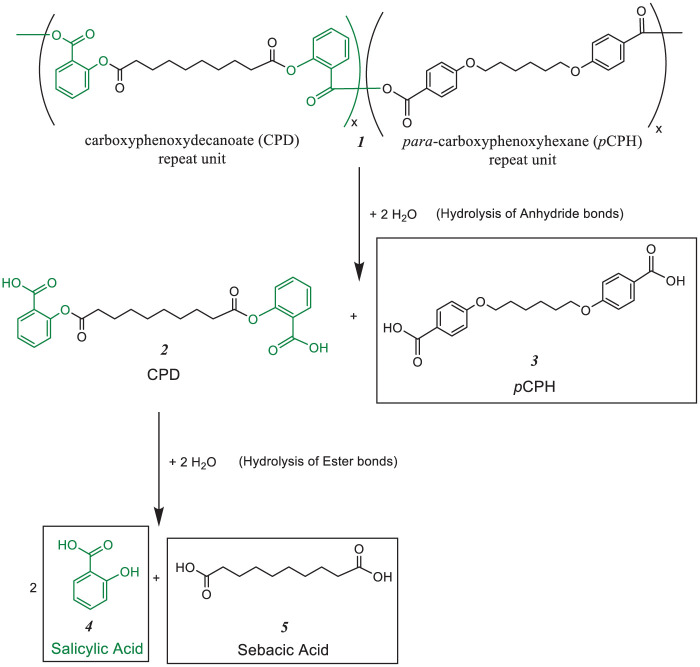
Hydrolysis of 50:50 CPD:*p*CPH copolymer (1). The anhydride bonds are hydrolyzed to form the CPD and CPH diacids (2 and 3). The ester linkages of CPD diacid (2) are further hydrolyzed to salicylic acid (4) and sebacic acid (5).

For this work, copolymers containing 50% CPD were chosen as they possessed the necessary physical properties, that is, ease of handling.^16^ Specifically, we evaluated the pH-sensitivity of copolymer (1) comprising ether-containing carboxyphenoxyhexane (pCPH) (3) and carboxyphenoxydecanoate (CPD) (2), noting that the ester bonds are hydrolyzed to salicylic acid (SA) (4) and sebacic acid (5) as outlined in Scheme 1. This manuscript describes the hydrolytic degradation of fibers comprised from 50:50 CPD:*p*CPH in media with pH values ranging from pH 6 to 9. This pH range was chosen as it covers the pH values found in the GCF (for periodontal disease) as well as in the gastrointestinal tract (potentially for intestinal delivery). The hydrolysis rates of the polymer fibers were examined over a 10-day time period to determine the influence of pH on polymer hydrolysis.

## Materials and methods

### Materials

#### Preparation of copolymer fibers

The copolymers and fibers were prepared as previously described.^16,17^ In brief, the copolymers were extruded using a Kayeness Dynisco 2000 as a vertical extruder with a 1-mm-diameter orifice. Polymer fibers were melt-drawn using a spindle with variable speed control. Fibers were produced at draw ratios of ~5:1–8.5:1. The polymer fibers described in this study were extruded at 65 °C under a pressure of 8.6 kN, corresponding to a shear force of ~8.83 g/m^2^. The fibers were spun at an average flow rate of 0.027 g/min, using a draw rate of 7.91 mm/s or a draw ratio of 6.25:1. The average 50:50 CPD:*p*CPH fiber diameter was 0.20 mm, as measured by using Vernier Calipers (Mitutoyo Corporation).

#### Preparation of phosphate buffered saline

Potassium dihydrogenphosphate (KH_2_PO_4_ – Aldrich, USA; 13.6 g) was added to potassium hydrogenphosphate (K_2_HPO_4_ – Aldrich, USA; 17.4 g) in a 1-L volumetric flask. Approximately 900 ml of distilled water were added to the flask and the solution was swirled until all the solids dissolved. Four N NaOH (Fisher, USA) or 1 N HCl (Corco Chemical Co., Fairless Hills, PA, USA) solutions were used to adjust the pH of the buffer solutions, as measured by a model 47 Mini pH-meter (VWR Scientific, Inc., San Francisco, CA, USA).

### Methods

#### Determination of salicylic acid solubility

Previously described protocols were used to determine solubility.^14,19^ An excess of salicylic acid (SA) was added to 10 ml of PBS at different pH values. The saturated solutions were titrated to the appropriate pH using 4 N NaOH and 1 N HCl, as the pH levels became more acidic (except the solution at pH 7) with the addition of excess SA. Saturated solutions were stored in a controlled environment incubator/shaker (New Brunswick Scientific Co., Inc., Edison, NJ) at 37 °C and 60 rpm for 48 h. The pH of all saturated solutions was checked prior to UV analysis and adjusted as necessary. Solutions requiring either additional titration or those that were no longer saturated, and therefore requiring the addition of more SA, were stored for an additional 48 h. The samples were diluted, filtered through 0.45-*m*m PTFE syringe filters (Whatman, Clifton, NJ, USA) and analyzed on a DU® Series 500 Spectrophotometer (Beckman Instruments, Fullerton, CA, USA) at a wavelength of 228 nm (a maximum on the SA absorbance spectrum).

#### High performance liquid chromatography

Salicylic acid concentrations in the degradation media were determined by high performance liquid chromatography (HPLC) on a Perkin Elmer Series 200 LC system equipped with a Discovery RP Amide C16 column operated at room temperature, Applied Biosystems ultraviolet detector l = 334 nm), Series 200 LC pump and IS 200 autosampler (typically 1–2 µl injection volumes). A Dell Optiplex GX110 computer running PE TurboChrom 4 software was used for data collection and processing and to automate the analysis via PE-Nelson 900 Interface and 600 Link. The eluting solvent was a 75/25 mixture of water (Fisher Scientific) and acetonitrile (Fisher Scientific) containing 0.1% trifluoroacetic acid (TFA) (Fisher Scientific) at a flow rate of 1.0 ml/min. Samples were filtered using 0.45 mm PTFE syringe filters prior to column injection.

#### Scanning electron microscopy

The copolymer samples were first affixed to the sample holders using non-conducting adhesive tabs (Electron Microscopy Sciences, Fort Washington, PA, USA). An amalgam of Au-Pd was then sputtered onto the copolymer samples (25 nm thickness) with a Baltec SCD 004 Sputter Coater. The scanning electron microscope (SEM) is an AMRAY 1830 I (AMRAY, Inc., Bedford, MA, USA) that uses FlashBus FBG 4.2 on Windows 2000 software to capture the images.

#### Copolymer fiber degradation

Approximately 3.3 mg of polymer fiber were placed in each 20-ml scintillation vial containing 5 ml of buffer solution at the appropriate pH, and stored in a Series 25 Controlled Environment Incubator Shaker at 37 °C and 60 rpm. The spent degradation media was decanted at predetermined intervals and replaced with 5 ml of fresh media. The spent degradation media was then analyzed by HPLC. Samples were prepared in triplicate.

## Results and discussion

Building upon the many polyanhydride studies, we focus on polyanhydrides that contain hydrolyzable ester bonds as well as non-hydrolyzable ether bonds.^20^ Comonomers with ether linkages are utilized to enhance the thermo-mechanical properties of the copolymers, where ester linkages lead to SA release. The 50:50 CPD:*p*CPH copolymer (1) undergoes hydrolysis of the anhydride bonds to yield *para*-carboxyphenoxyhexane (*p*CPH) (3) and an intermediate, carboxyphenoxydecanoate (CPD) (2) diacid. The ester bonds in the CPD diacid are ultimately hydrolyzed to ultimately give two equivalents of salicylic acid (SA) (4) and one equivalent of sebacic acid (5) as the final degradation products, as outlined in Scheme 1.

### Role of pH on salicylic acid solubility

**Figure 1.**
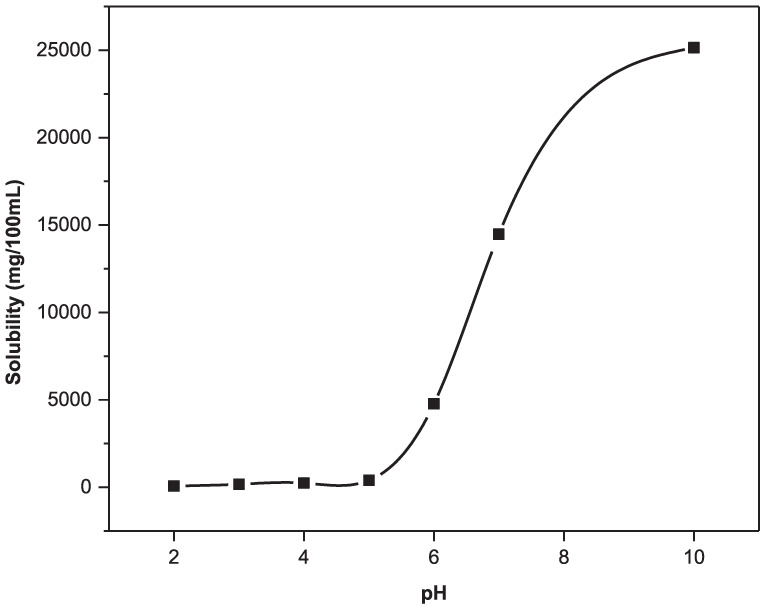
Solubility profile of salicylic acid (4) in phosphate buffered solutions ranging from pH 2 to 10. For these studies, an excess of salicylic acid (4) was placed into vials with specific pH values, and stored at 37 °C with shaking for 48 h. The solutions were filtered, diluted, and analyzed at the absorbance maximum for salicylic acid (4) at 228 nm.

As the degradation products of copolymer 1 are all carboxylic acids (2–5), increasing the degradation media pH to more basic pH is expected to enhance the solubility of the degradation products. The solubility of salicylic acid (4), the bioactive component of the copolymer, was examined as a function of pH (Figure 1). Increasing the pH (i.e. increased basicity) of the solution did indeed enhance the solubility of SA.

The solubility profile has a sigmoidal shape, similar to that for the solubility profiles of both sebacic acid and *para*-carboxyphenoxypropane, as determined by other researchers.^20–22^ SA was only slightly soluble in acidic media; at pH 2 the solubility was roughly 0.67 mg/ml. When the solution was neutral, at pH 7, SA solubility increased by more than 200 times to about 145 mg/ml. When the solution was basic, at pH 10, the solubility of SA had reached ~252 mg/ml; the solubility of SA had increased by almost 400 times that at pH 2.

### Role of pH on copolymer degradation

A recent study investigated how pH conditions influenced degradation of SA-based poly(anhydryide-ester) disks.^23^ In that work, complete SA release was achieved at more basic conditions (pH 8–9, whereas acidic conditions (pH 2–4) minimized degradation of poly(anhydride-esters). In this study, we similarly investigated copolymers of SA-based copolymer fibers comprising both ester and ether linkages.

**Figure 2.**
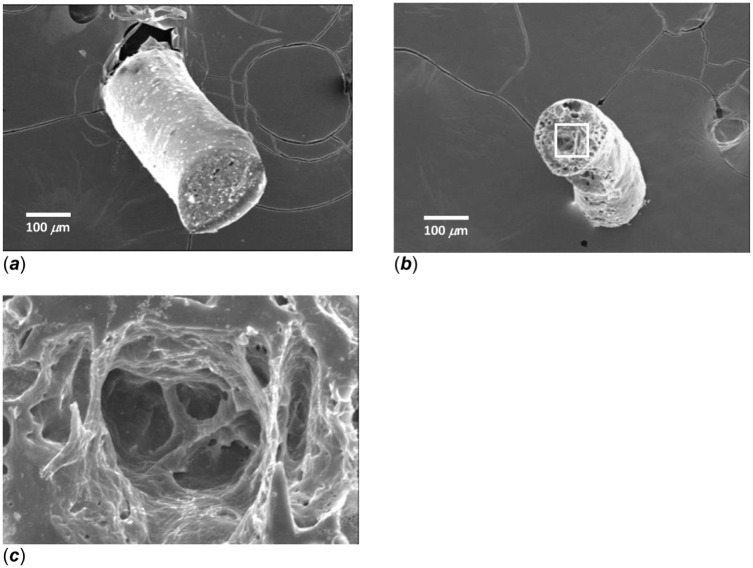
SEM micrograph of 50:50 CPD:*p*CPH copolymer fibers (a) prior to hydrolysis and (b) after 24 h of hydrolysis in PBS at pH 7 (100×). (c) Interconnected porous structure found in 50:50 CPD:*p*CPH copolymer fibers after 24 h of hydrolysis in PBS at pH 7 (500× view of highlighted region in (b)). SA: salicylic acid.

Previous work has demonstrated that when copolymers comprised of ester and ether linkages (ie, CPD:*p*CPH) degrade, pore formation begins at the surface, ultimately moving to the interior region of the copolymer matrix.^16^ As an example, after 24 h of degradation in pH 7 media, the 50:50 CPD:*p*CPH fibers have a complex porous structure within the interior (Figure 2). Degradation products must diffuse through this interconnecting network of pores (Figure 2(c)). SA release at this point is no longer a function of only polymer hydrolysis, but also becomes a function of SA diffusivity through the porous network.

Degradation product solubility is one of the key driving forces for salicylate-based polyanhydride hydrolysis, along with bond lability towards hydrolysis. Enhancing the solubility of the degradation products, by increasing the pH of the degradation media, should then increase the rate at which the 50:50 CPD:*p*CPH copolymers fibers are hydrolyzed. Thus, we evaluated the impact of pH on the copolymer degradation, where the fibers are comprised of ester and ether linkages.

**Figure 3.**
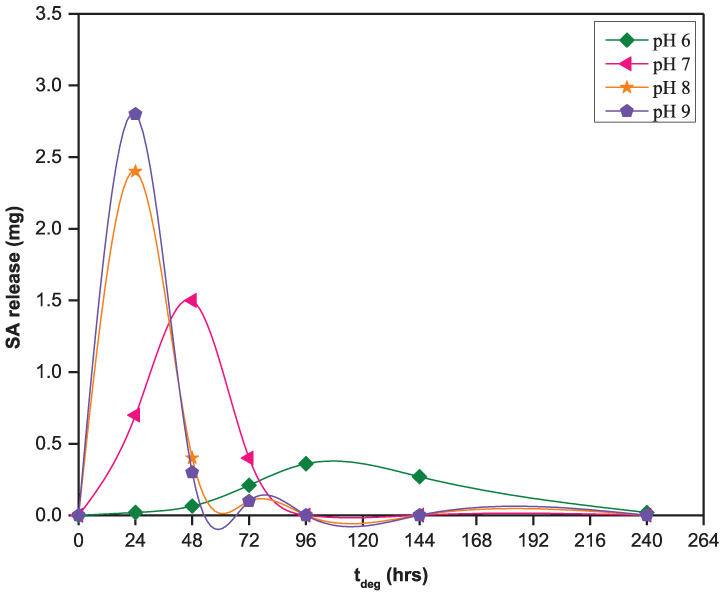
SA release from 50:50 CPD:*p*CPH copolymer fibers in phosphate buffered solutions at pH levels from 6 to 9. SA: salicylic acid.

The release of SA from the 50:50 CPD:*p*CPH copolymer fibers in the pH range from 6 to 9, the pH range relevant for oral and wound care, is presented in Figure 3. Over this pH range, SA release is proportional to the increase in pH of the degradation media. After 24 h of hydrolysis, the 50:50 CPD:*p*CPH fibers were releasing 0.020 mg/day of incorporated SA at pH 6, 0.70 mg/day at pH 7, 2.4 mg/day at pH 8, and 2.9 mg/day at pH 9. After 10 days of hydrolysis at pH 6, the 50:50 CPD:*p*CPH fibers were releasing 0.020 mg/day of incorporated SA. During the same time period at pH 7–9, the 50:50 CPD:*p*CPH fibers were completely hydrolyzed (Figure 3).

**Figure 4.**
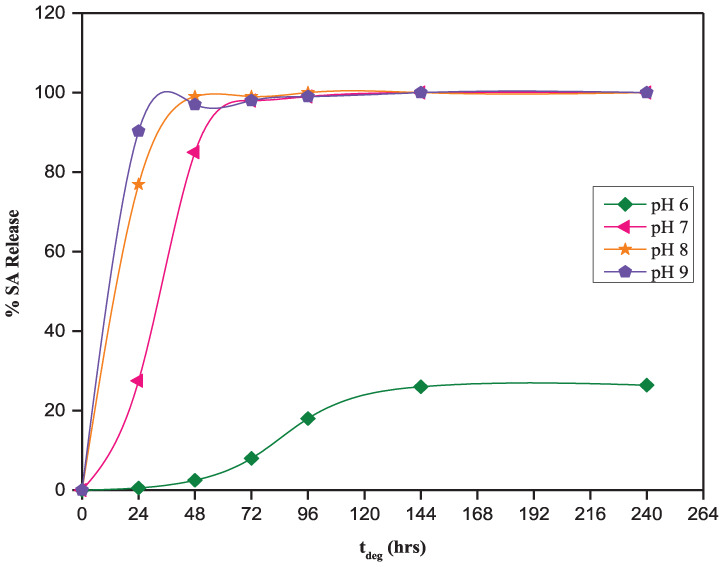
Cumulative salicylic acid release from 50:50 CPD:*p*CPH copolymer fibers in phosphate buffered solutions at pH 6–9.

The solubility of SA in media at pH 6–9 is sufficient to solubilize the degradation products of the 50:50 CPD:*p*CPH fibers, as all sets of fibers are releasing detectable amounts of SA within the first 24 h of exposure to degradation media (Figure 4). Increasing the pH of the degradation media, from pH 6 to 9, does serve to increase the rate at which the 50:50 CPD:*p*CPH fibers are hydrolyzed, as expected from the solubility profile of SA (Figure 1). After 24 h, the 50:50 CPD:*p*CPH fibers had released only 0.54% of the SA incorporated into the polymer matrix at pH 6. In contrast, at pH 7, the polymer fibers had released 28% of the SA, at pH 8 the fibers had released 77% of the SA and at pH 9, the polymer fibers had released 90% of the SA.

This data is in agreement with the solubility data (Figure 1), where SA is 200 times less soluble in acidic pH than at neutral pH and nearly 400 times less soluble than at basic pH. Because the degradation products are carboxylic acids, they are more soluble in basic media. Thus, hydrolysis of the 50:50 CPD:*p*CPH fibers proceeded more quickly as pH of the degradation media was increased. After 10 days of hydrolysis, the 50:50 CPD:*p*CPH fibers had only released 26% of the incorporated SA at pH 6, whereas, the 50:50 CPD:*p*CPH fibers had essentially undergone complete hydrolysis in more basic media (pH 7–9) in the same time period.

## Conclusions

The 50:50 CPD:*p*CPH fibers were degraded in phosphate buffer solutions over a relevant range of pH values to determine the effect pH had on the copolymer hydrolysis. Over the pH range from pH 6 to 9, increasing the pH of the degradation media increases the rate of anhydride hydrolysis of the 50:50 CPD:*p*CPH fibers. We observed that at more basic conditions (~pH 9), the copolymer fibers are nearly completely hydrolyzed in 6 days, though remnants of the fibers remain in the vials to release minimal amounts of SA.

To be useful in periodontal applications, the 50:50 CPD:*p*CPH fibers must hydrolyze, and thereby release salicylic acid over a pH range of 6–9. Furthermore, the copolymer fibers should remain within the periodontal pocket for a period of at least 1 week under the above specified conditions.^24^ Considering the pH-sensitivity of the biodegradable copolymers and their adaptation to environmental pH changes, these pH-responsive polymers are ideal for periodontal drug delivery. In addition, these polymers may provide a solution for advanced drug delivery in other pH-specific environments, like acidic tumors or the gastrointestinal tract, while effectively degrading into bioactive compounds.
